# HNA-3a and HNA-3b antigens among 9 ethnic populations and the Han population in Southwest China

**DOI:** 10.1186/s12967-018-1447-1

**Published:** 2018-03-14

**Authors:** Guo-Jin Ou, Pin-Can Su, Hao Yu, Xin Ji, Fan Liu, Sheng-Lan Wang, Yu-Jie Kong, Ling Li, Jue Wang, Zhong Liu, Willy Albert Flegel

**Affiliations:** 1 Clinical Blood Transfusion Research Center, Institute of Blood Transfusion, CAMS & PUMC, No. 26 Hua-Cai Road, Chenghua District, Chengdu, 610052 Sichuan China; 2 Transfusion Medicine Research Department, Yunnan Kunming Blood Center, Kunming, China; 3 Blood Center of the Liangshan Autonomous Region, Sichuan, China; 4 Department of Transfusion Medicine, NIH Clinical Center, National Institutes of Health, Bethesda, MD USA; 5 Key Laboratory of Transfusion Adverse Reactions, CAMS, Chengdu, Sichuan China

**Keywords:** HNA-3 antigens, TRALI, Gene polymorphism, PCR-SBT genotyping

## Abstract

**Background:**

Human neutrophil antigen 3 (HNA-3) is encoded by the SLC44A2 gene. Antibodies against HNAs can cause severe, often fatal, transfusion reactions, known as transfusion-related acute lung injury, and neonatal neutropenia. We explored the 2 common HNA-3 variants in 9 ethnic populations residing in Sichuan and Yunnan provinces of China as compared to the Han population.

**Methods:**

We genotyped for *SLC44A2* (rs2288904) by polymerase chain reaction sequence-based typing among blood donors, for a total of 2206 individuals in Yunnan and 376 in Sichuan.

**Results:**

The SLC44A2*02 allele (HNA-3b antigen) frequency varied between 0.24 and 0.33 for all 9 ethnic populations in Yunnan, including Zhuang, Derung, Hani, Lisu, Bai, Miao, Dai, Naxi, and Yi. Specifically, the Yi ethnicity did not present an unusually great SLC44A2*02 frequency at any of the 4 locations examined in Yunnan. Except of the Yi ethnicity in Sichuan (0.40), the Han ethnicity, as the majority population group, had the greatest SLC44A2*02 frequency with 0.39 in Yunnan and 0.35 in Sichuan.

**Conclusion:**

The ethnic populations in Southwest China are not at an increased risk for anti-HNA3a compared to the Han population, with the possible exception of Yi in Sichuan. Our data, however, corroborated the known high prevalence of SLC44A2*02 in Han populations. Hence, the Han populations in Yunnan, Sichuan and elsewhere in China are at a comparatively great risk for developing HNA-3a antibodies.

## Background

The human neutrophil antigen (HNA) system was established in 1998 by an International Society of Blood Transfusion (ISBT) Working Party [1, 2]. Nine HNAs have been identified, i.e., HNA-1a, -1b,-1c, -3a, -3b, -4a, -4b, -5a, and -5b. HNA-1, -4, and -5 are expressed on glycoproteins CD16b (FcgR3b), CTL2, CD11b/18 (Mac-1, CR3, αMβ2-integrin), and CD11a (LFA-1, αMβ2-integrin), respectively [3]. Most HNA antigens are caused by single-nucleotide polymorphisms (SNPs) in the underlying genes, determining the different allelic forms.

HNA-3a and HNA-3b were identified in 1964 using antibodies from multiparous women that agglutinated leukocytes [4]. In 2010, the *SLC44A2* gene has been identified to express the HNA-3 antigens occurring on the choline transporter-like protein 2 (CTL2) [5]. The *SLC44A2* gene has the rs2288904 single-nucleotide polymorphism, encoding the common HNA-3 protein variants Arg154 (HNA-3a) and Gln154 (HNA-3b), whichis used for *SLC44A2* (rs2288904) genotyping.

HNA-3 is particularly important for the pathophysiology of transfusion-related acute lung injury (TRALI) and neonatal alloimmune neutropenia [6–8]. Individuals who lack the HNA-3a or HNA-3b antigens can be immunized and produce antibodies when exposed to the cognate antigen via blood transfusion or pregnancy. The HNA-3a antigen is particularly important owing to the association between anti-HNA-3a and TRALI [9].

Only a few cases of TRALI have been reported in China [10–12]. In a retrospective study among pediatric surgical patients, Xing et al. identified TRALI in two of 1495 transfusion cases [10]. Hence, the incidence of TRALI was higher than reported previously, which ranged from 1:202,673 in plasma to 1:2,527,437 in red blood cells [13]. Blood components from female donors are still used by hospitals in China. Anti-HLA were found among 5.6% of nulliparous women [14], while 26.6% of female blood donors in China have a history of pregnancies. Despite the large numbers of plasma and platelet components transfused, the lack of awareness and diagnostic standards for TRALI, may lead to the failure of recognizing TRALI in China.

HNA-3 antigen frequencies vary among populations and countries. Population studies have shown that 13–19% [15, 16] of Chinese Han individuals are negative for the HNA-3a antigen and are at risk for alloimmunization and development of anti-HNA-3a. There are 56 ethnic populations recognized in China. The Yi population of Xichang in the south of Sichuan province had the greatest frequency of blood donors at risk of harboring anti-HNA-3a [15] ever reported. To further explore the populations in Southwest China, we determined the frequencies of the alleles encoding HNA-3a (SLC44A2*01) and HNA-3b antigens (SLC44A2*02) in 9 ethnic populations residing in Sichuan and Yunnan provinces of China as compared to the Han population.

## Methods

### Blood samples

**Fig. 1:**
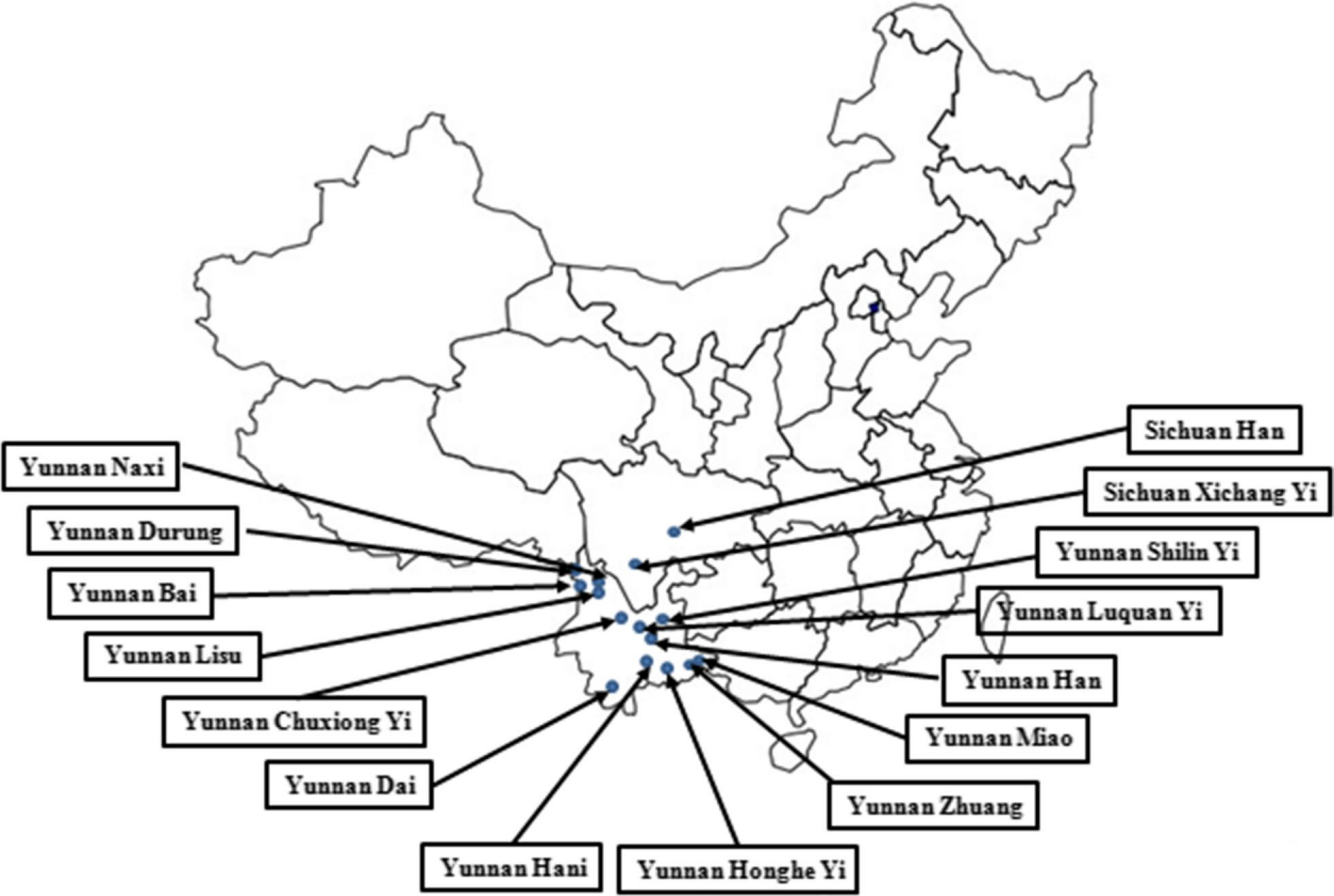
The locations of the populations in Southwest China. Blood samples came from 9 ethnicities of the minority population groups in Yunnan province. Additional blood samples from the Yi ethnicity in Sichuan and the majority population group of Han ethnicity in Yunnan and Sichuan were collected for comparison

A total of 2582 samples were included in this study. Among them, 232 blood samples were obtained from Chinese Sichuan Han individuals collected at the Sichuan branch of the Chinese Marrow Donor Program (CMDP), and 144 Chinese Sichuan Xichang Yi blood donors collected at the Liangshan blood center. The Xichang Yi samples included the 100 samples previously reported samples by Chen et al. [15]. Another 2206 Yunnan individual samples including 196 Zhuang, 184 Derung, 190 Hani, 254 Lisu, 190 Bai, 209 Miao, 126 Dai, 187 Naxi, 83 Shilin Yi, 122 Luquan Yi, 120 Honghe Yi, 153 Chuxiong Yi and 192 Yunnan Han populations were collected at the Kunming blood center in Yunnan province (Fig. 1). The ethnicity were self-declared by the blood donors.

To ensure the correct ethnic background, we collected all blood samples in the local ethnic autonomous counties where the respective ethnic blood donors represented the vast majority of the population. Also, the volunteers were enrolled only after we carefully checked their identity cards documenting their residency in the county. Finally, blood donors were asked by our staff for their parents’ ethnicity; unless both parents belonged to the local ethnicity, the blood donors were excluded from the study. All samples were collected after obtaining informed consent and the study was approved by the Ethics Committee of the Institution of Blood Transfusion (IBT), CAMS & PUMC, and Kunming blood center in Yunnan province.

### Genotyping

Genomic DNA was extracted from venous blood samples (TIANamp Blood DNA Kit; Tiangen, Beijing, China) at 20–50 ng/µL concentration. The *SLC44A2* genotype (rs2288904) was examined by polymerase chain reaction (PCR)–sequence-based typing. The primers used to amplify a fragment of 291 bp containing the rs2288904 SNP from genomic DNA were as follows: forward, 5′-GGGCAGTGGCAGTGTACTA-3′, and reverse, 5′-CATGCCCATCCTCATAGGTCG-3′. The PCR mix contained 1 µL of DNA and 5 µL of master mix (GoTaq Green; Promega, Madison, WI, USA) plus 5 µM primers for a total volume of 10 µL. Thermal cycling conditions were as follows: 96 °C for 5 min; 35 cycles at 96 °C for 30 s, 60 °C for 30 s, 72 °C for 45 s; and a final extension at 72 °C for 7 min. The PCR products (3 µL) were resolved on 2% agarose gels and visualized under UV with ethidium bromide, followed by purification of PCR products (7 µL) using Exo I and FastAP enzymes (Thermo; Vilnius, Lithuania). Sequencing was conducted with 1 µL of purified PCR products (BigDye Terminator Ready Reaction v3.1 kit; Applied Biosystems, Foster City, CA, USA) using the reverse primer (ABI 3730 DNA Sequencer; Applied Biosystems). Standard thermal cycling conditions for sequencing reactions were used: 96 °C for 1 min; 25 cycles at 96 °C for 10 s, 50 °C for 5 s, and 60 °C for 4 min.

### Statistical analysis

SLC44A2*01 and SLC44A2*02 alleles observed in the 3 genotypes were counted and the allele frequencies calculated (Table 1). The Chi square test was used to exclude the possibility of deviations from the Hardy–Weinberg equilibrium in all 15 populations tested. Comparisons of allele frequencies between populations were performed using the Chi square test or Fisher’s exact test (when the minimum expected frequency was less than five).

**Table 1:** *SLC44A2* allele distribution and HNA-3 antigens predicted by genotype

Blood donors					Genotypes			*SLC44A2* allele frequency		Hardy–Weinberg		Chance of being exposed to an alloimmunization risk against HNA-3a
Population group	Ethnicity	Location	Province	n	HNA-3a/a	HNA-3a/b	HNA-3b/b	HNA-3a	HNA-3b	χ^2^	*P*	
Minority	Yi	Honghe	Yunnan	120	61 (51%)	53 (44%)	6 (5%)^a^	0.73	0.27^b,c^	1.67	0.2	0.068
		Chuxiong	Yunnan	153	82 (54%)	58 (38%)	13 (8%)^a^	0.73	0.27^b,c^	0.36	0.55	0.068
		Luquan	Yunnan	122	55 (45%)	56 (46%)	11 (9%)^a^	0.68	0.32^d^	0.37	0.54	0.092
		Shilin	Yunnan	83	39 (47%)	33 (40%)	11 (13%)	0.67	0.33^d^	0.88	0.35	0.097
		Xichang	Sichuan	144	57 (40%)	59 (41%)	28 (19%)	0.60	0.40	3.06	0.08	0.134
	Zhuang	Wenshan	Yunnan	196	115 (59%)	69 (35%)	12 (6%)^a^	0.76	0.24^b,c^	0.15	0.70	0.054
	Derung	Nujiang	Yunnan	184	107 (58%)	62 (34%)	15 (8%)^a^	0.75	0.25^b,c^	1.89	0.17	0.059
	Hani	Honghe	Yunnan	190	119 (63%)	59 (31%)	12 (6%)^a^	0.78	0.22	1.56	0.21	0.046
	Lisu	Lijiang	Yunnan	254	146 (57%)	87 (34%)	21 (8%)^a^	0.75	0.25^b,c^	2.34	0.13	0.059
	Bai	Dali	Yunnan	190	95 (50%)	79 (42%)	16 (8%)^a^	0.71	0.29	0.01	0.94	0.077
	Miao	Wenshan	Yunnan	209	101 (48%)	90 (43%)	18 (9%)^a^	0.70	0.30^d^	0.11	0.74	0.082
	Dai	Xishuangbanna	Yunnan	126	62 (49%)	51 (40%)	13 (10%)	0.69	0.31	0.27	0.60	0.087
	Naxi	Lijiang	Yunnan	187	85 (45%)	86 (46%)	16 (9%)^a^	0.68	0.32	0.78	0.38	0.092
Majority	Han	Yunnan	Yunnan	192	67 (35%)	102 (53%)	23 (12%)	0.61	0.39^d^	2.83	0.09	0.129
	Han	Sichuan	Sichuan	232	94 (41%)	113 (49%)	25 (11%)	0.65	0.35	1.09	0.30	0.107

Differences were significant when *P *< 0.05

^a^The genotype frequency significantly differs compared to Sichuan Yi (Chi square test)

^b^The allele frequency significantly differs compared to Sichuan Yi (Chi square test)

^c^The allele frequency significantly differs compared to Sichuan Han (Chi square test)

^d^The allele frequency significantly differs compared to Yunnan Zhuang (Chi square test)

The chance of being exposed to HNA-3a by non-leukocyte-reduced blood components, while lacking this antigen, was calculated as (2 × SLC44A2*01 × SLC44A2*02 + SLC44A2*01^2^) × SLC44A2*02^2^; the chance of being exposed to HNA-3b while lacking that antigen was calculated similarly. *P *< 0.05 was considered significant.

## Results

We determined the frequencies of the 2 main *SLC44A2* alleles, encoding the 2 HNA-3 antigens, in 9 ethnic populations residing in the Sichuan and Yunnan provinces of China (Fig. 1). For comparison, the majority population group of Han ethnicity in Yunnan and Sichuan were tested, along with an enlarged sample for the Yi ethnicity in Sichuan.

### Ethnic populations in Southwest China

The Yi ethnicity at any of the 4 locations in the Yunnan province did not present with an unusual great frequency of the SLC44A2*02 (HNA-3b) allele (Table 1). The 8 other ethnic populations in Yunnan, including Zhuang, Derung, Hani, Lisu, Bai, Miao, Dai, and Naxi, varied in SLC44A2*02 (HNA-3b) allele frequencies between 0.24 and 0.32 similar to the variability between 0.27 and 0.33 observed for Yi in Yunnan.

### Han population in Southwest China

The Han population of Yunnan had the greatest frequency of the SLC44A2*02 (HNA-3b) allele with 0.39 (Table 1), barely short of Yi in Sichuan with 0.40 in this study and 0.41 as reported previously [15].

### Rate of exposure to the HNA-3a antigen

The data allowed to calculate the chance of being exposed to the HNA-3a or HNA-3b antigen, while lacking the cognate antigen; each transfusion may pose an alloimmunization risk when non-leukocyte-reduced blood components are use (Table 1). These rates differed substantially but would strictly apply for transfusions among members of a given ethnicity, such as in directed transfusions, or to transfusions among Han majority population. Of note, the rate was consistently greatest for the Han population (Table 1), in accordance with all previously published *SLC44A2* genotype data [15].

## Discussion

The Yi population in the south of the Sichuan province had the greatest frequency of blood donors at risk of harboring anti-HNA-3a reported so far [15]. This finding prompted us to explore the alleles encoding HNA-3a (SLC44A2*01) and HNA-3b antigens (SLC44A2*02) in 9 ethnic populations residing in the immediately adjacent Yunnan province (Fig. 1). The SLC44A2*02 allele frequency varied between 0.24 and 0.33 for all 9 ethnic populations in Yunnan. Specifically, the Yi ethnicity did not present an unusually great SLC44A2*02 frequency at any of the 4 locations examined in Yunnan. Assuming the previously reported [15] unusual great frequency of the SLC44A2*02 among Yi in Sichuan represented a random fluctuation, we conclude that the 9 ethnic populations in Southwest China are not at an increased high risk for anti-HNA3a compared to the Han population.

The SLC44A2*02 frequencies in all populations examined in this study were significantly higher than in European [16–19], other Caucasian [20, 21], and American populations [22]. Our data corroborated the known high prevalence of HNA-3b in Han populations [23, 24]. Hence, the Han populations in Yunnan, Sichuan and elsewhere in China are at a higher risk for developing HNA-3a antibodies compared to most populations outside of China [16–23]. We confirmed that the HNA-3b frequencies of Yi and Han residing close to each other in Sichuan were almost identical. Intermarriage among Yi and Han may have contributed and could have been more prevalent in Sichuan compared to the adjacent Yunnan province. The number of individuals per ethnic population ranged, with 1 exception, from 120 to 254 (Table 1) and should be large enough to allow an approximation. Particularly, the *SLC44A2* (HNA-3a and HNA-3b) alleles were in Hardy–Weinberg equilibrium in all 15 locations tested representing 10 distinct ethnic populations, making stochastic sampling errors unlikely.

The prevalence of individuals in China, who are at risk of developing anti-HNA-3a, may be considered for clinical decisions, such as excluding plasma donations by previously pregnant females, because TRALI is currently the most common cause of transfusion-related deaths in Western health care systems, and approximately 80% of reported TRALI cases are associated with the transfusion of blood products containing unsuspected leukocyte-specific alloantibodies [15], mainly anti-HNA or anti-HLA. A retrospective study of 36 TRALI cases showed that HNA antibodies, especially anti-HNA-3a, accounted for 10 of 12 HNA antibody-mediated reactions and 6 of 10 fatalities, including one that occurred after the transfusion of red blood cells. Antibodies with specificity against HNA-1a and HNA-2a were only detected in 2 of 36 TRALI cases [7]. HNA-3a alloantibodies are prone to cause severe and often fatal clinically relevant complications [25–27]. Individuals expressing only the HNA-3b antigen can form antibodies against the HNA-3a antigen, whereas individuals expressing only the HNA-3a antigen can form antibodies against the HNA-3b antigen.

Only the Japanese population (37% for HNA-3b) [28], has been reported to have HNA-3b frequencies similar to Chinese populations. Other Asian populations, such as Thai (31%) [29, 30] and Asian Indian (24%) [22], resemble the Caucasian prevalences, which interestingly was also reported in a large cohort of 493 Han individuals in Guangzhou (26%) [24]. Anti-HNA-3a were found in 0.23% of previously pregnant women, reflecting an immunization rate of 7%, in the population of Germany where 4% can be expected to be HNA-3a negative.

Compared to any Western population [16–21], the chance of being exposed to an alloimmunization risk against HNA-3a is 2 times greater in ethnic populations, but 2–3 times greater in the Han population. Assuming a 0.23% prevalence similar to Western studies, up to 1,000,000 women in China may be at risk of HNA-3a alloimmunization. Among 14,000,000 blood donors in 2016 female donors comprised 30%, of whom 10,000 may harbor anti-HNA-3a (14 mio × 30% × 0.23%). However, anti-HNA-3a causing TRALI has been rarely reported in China [10]. If anti-HNA-3a and TRALI in China were truly less common than in Western settings, this should prompt research for the biologic reason, such as possible protective HLA haplotypes prevalent in China but lacking in Western populations; this research may also address the unexplained observation why the immunization rate for anti-HNA-3b (0.7%) is so much smaller than for anti-HNA-3a (7%) [31]. If anti-HNA-3a and TRALI are more common, as they may be in China, improved hemovigilance in combination with prevention strategies should be evaluated and implemented similar to Western health care systems. Also, detection of HLA or HNA antibodies in blood donors, especially in women with childbearing history, can definitely improve blood transfusion safety in China.

## Conclusion

More studies in Chinese populations may be worthwhile to evaluate the risk and possible need to prevent the occurrence of TRALI. The risk of TRALI caused by HNA-3a antibodies can be reduced by excluding female blood donors from plasma and platelet donations or any donor who is homozygous for the SLC44A2*02 allele in China [15].

## Abbreviations

TRALI: transfusion-related acute lung injury
HNA: human neutrophil antigen
CMDP: Chinese Marrow Donor Program
ISBT: International Society of Blood Transfusion
